# Tellurium-Doped Silanised Bioactive Glass–Chitosan Hydrogels: A Dual Action for Antimicrobial and Osteoconductive Platforms

**DOI:** 10.3390/polym17121651

**Published:** 2025-06-13

**Authors:** Matteo Bergoglio, Ziba Najmi, Federica Ferla, Alessandro Calogero Scalia, Andrea Cochis, Lia Rimondini, Enrica Vernè, Marco Sangermano, Marta Miola

**Affiliations:** 1 Dipartimento Scienza Applicata e Tecnologia, Politecnico di Torino, Corso Duca degli Abruzzi 24, 10129 Torino, Italy; matteo.bergoglio@polito.it (M.B.); enrica.verne@polito.it (E.V.); marco.sangermano@polito.it (M.S.); 2 Department of Health Sciences, Center for Translational Research on Autoimmune and Allergic Diseases—CAAD, Università Del Piemonte Orientale (UPO), 28100 Novara, Italy; ziba.najmi@uniupo.it (Z.N.); federica.ferla@uniupo.it (F.F.); andrea.cochis@med.uniupo.it (A.C.); lia.rimondini@uniupo.it (L.R.)

**Keywords:** bioactive glasses Te-reinforced, bio-based hydrogel, photopolymerisation, composite hydrogels

## Abstract

UV-cured methacrylated chitosan (MCHIT) hydrogels were achieved in the presence of silanised tellurium-doped silica bioactive glass (BG-Te-Sil) to produce an antimicrobial and osteoconductive scaffold for tissue engineering applications. Methacrylation of chitosan enabled efficient crosslinking, and the curing process was evaluated by means of Fourier-transform infrared spectroscopy (FTIR) and photorheology analyses. Compressive testing on crosslinked hydrogels showed that the silanised, bioactive, doped glass increased the hydrogel’s elastic modulus by up to 200% compared to unreinforced controls. Antibacterial assays against *Staphylococcus aureus* ATCC 43300 revealed a significant (*p* < 0.05) reduction in bacterial metabolic activity for hydrogels containing 50 wt% of the Te-doped bioactive glass. In vitro cytocompatibility with human bone-marrow mesenchymal stem cells demonstrated sustained viability and uniform distribution at 72 h (live/dead staining, AlamarBlue). Under H_2_O_2_-induced oxidative stress, reinforced hydrogels downregulated pro-inflammatory genes (TNF-α, IFN-γ, IL-1β, and PGES-2). These results suggest that the presence of the silanised bioactive glass can significantly enhance mechanical stability, antibacterial properties, and anti-inflammatory responses without affecting cytocompatibility, making these hydrogels promising for tissue engineering applications.

## 1. Introduction

Hydrogels are three-dimensional, crosslinked structures with a highly hydrophilic nature, capable of retaining large amounts of water or biological fluids—often exceeding 90%—while maintaining their structural integrity. Due to their soft, skin-like properties, hydrogels have been extensively employed in tissue engineering (TE) applications [1,2,3,4,5,6,7,8]. When they are immersed in a biological fluid, they create a hydrated microenvironment that mimics the dynamic extracellular matrix (ECM) found in native tissues [9,10,11,12].

Bio-based and natural polymers are particularly noteworthy among hydrogel precursors due to their characteristics. Bio-based hydrogel properties are dependent on their composition and from what they are derived but they mainly present common advantages such as availability, biodegradability, low toxicity, and biocompatibility. Among the natural resources available to produce hydrogels, chitosan, a natural polymer derived from chitin, plays a crucial role in the category [13,14,15,16,17,18]. Chitin is commercially extracted from sources such as crab, shrimp, krill shells, and fungi, large amounts of which are byproducts of the food industry [19,20,21]. From chitin, chitosan can be derived through a process called deacetylation, where acetyl groups are removed using an alkaline treatment (typically with sodium hydroxide). Chitosan consists of randomly distributed β-(1→4)-linked D-glucosamine (deacetylated units) and N-acetyl-D-glucosamine (acetylated units). It is widely available, cost-effective, and possesses excellent biocompatibility and chemical reactivity [22]. For the explained reasons, chitosan can be an optimal starting point for hydrogel production. However, natural polymers like chitosan often require treatment and functionalisation before photopolymerisation.

Photopolymerisation is the most commonly used method to obtain chemically crosslinked hydrogels. This technique, which employs a photo-initiator, offers advantages such as rapid processing, mild reaction conditions, and the ability to tailor hydrogel geometries. The photopolymerisation of chitosan was studied extensively in our group, where chitosan was modified chemically to attach different chemical groups and enhance its properties, guaranteeing an optimal photocuring process or removing wastewater oil and toxic ions from water [23,24,25].

Incorporating bioactive glasses into hydrogel matrices represents a promising approach to enhance biocompatibility and introduce additional functionalities to the final crosslinked material. First synthesised by L.L. Hench, bioactive glasses (BGs) are osteoconductive and osteoinductive when placed in vivo. Their ceramic structure, primarily composed of CaO-P_2_O_5_-SiO_2_, facilitates the release of silica species with the formation of a silica layer of Ca^2+^, PO₄^3−^, and CO_3_^2−^ ions, ultimately leading to the formation of hydroxyapatite (HA). These properties improve the possibility of successful scaffold implantation [26,27].

Doping bioactive glasses with therapeutic ions further enhances their properties. Research has focused on doping BGs with ions such as silver (Ag^+^), gallium (Ga^3+^), zinc (Zn^2+^), copper (Cu^2+^), and cerium (Ce^3+^) to impart antibacterial, anti-inflammatory, or other beneficial effects [28]. Tellurium is another interesting ion that is barely considered in biology and biochemistry applications; this element and its derivatives demonstrate antibacterial, anti-inflammatory, and antitumoural properties, which are concentration-dependent [29]. In particular, it possesses strong antioxidant activity and antimicrobial effects against Gram-negative and Gram-positive bacteria [30,31,32].

In a previous study of our group, we designed and synthesised Te-doped bioactive glasses using a casting method to confer antibacterial and antioxidant properties while maintaining cytocompatibility. This was achieved by partially substituting SiO_2_ with TeO_2_ [33]. However, the casting method can be optimised by transitioning to low-energy techniques, such as sol-gel synthesis. Sol-gel synthesis is recognised as a versatile, reliable, and environmentally friendly technique. It offers simplicity, cost-effectiveness, and the ability to produce homogeneous materials with controlled porosity under mild chemical conditions [34,35,36,37]. However, the incorporation of bioactive glasses into a polymeric matrix can be challenging due to the lack of compatibility between the two components [38]. Different research methods have been employed to increase the compatibility between bioactive glasses and polymeric matrices [39]. Among them, surface modification and silanisation are suitable to meet the objective of improving the interface between the matrix and reinforcement phase [40,41,42,43]. In our group, we studied the surface modification through silanisation by using 3-(Trimethoxysilyl)propyl methacrylate (TMSPMA) as a silanising agent [44].

In this study, we designed a hydrogel system consisting of a polymeric matrix derived from methacrylated chitosan that was then reinforced with Te-doped bioactive glasses to impart additional properties to the final hydrogel. The methacrylation was successfully demonstrated, and a biological assessment was added to the mechanical and comprehensive tests performed on the obtained material.

## 2. Materials and Methods

### 2.1. Materials

Medium-molecular-weight chitosan (Mw = 190–310 KDa, N-deacetylation at 75–85% degree, MCHIT), acetic acid, methacrylic anidride (MA), 2-Hydroxy-4′-(2-hydroxyethoxy)-2-methylpropiophenone (98%), calcium nitrate tetrahydrate (Ca(NO_3_)_2_·4H_2_O), ammonium hydroxide (NH_4_OH), tetraethyl orthosilicate (TEOS), triethyl phosphate (TEP), sodium tellurite (Na_2_TeO_3_), 3-(Trimethoxysilyl)propyl methacrylate (98%, TMSPMA), ethanol (EtOH, 99%), hydrogen peroxide (H_2_O_2_), Tween 80, penicillin and streptomycin, Hematoxylin and Eosin were purchased from Sigma Aldrich (Milan, Italy). Lauria Bertani (LB) broth/agar was purchased from Thermo Fisher Scientific, Milan, Italy.

### 2.2. Synthesis of Bioactive Glasses

The bioactive glass was synthesised to be used as a reinforcement in the hydrogel structure using a sol-gel method. They were obtained as spherical particles doped with Tellurium and subsequently silanised. The synthesis involved mixing two different solutions into a 500 mL beaker under a fume hood during vigorous stirring. The first solution was composed of 46 mL of bi-distilled water, 30 mL of ethanol, and 17 mL of ammonium (NH_4_OH), while the second was composed of 11.2 mL of TEOS, the silica precursor, and 93 mL of ethanol. After the formation of silica particles due to the mixing of the two solutions, the dopant agent—0.84 mL of triethyl phosphate (TEP) (a phosphorous (P) precursor), 3.08 g of calcium nitrate tetrahydrate (Ca(NO_3_)_2_·4H_2_O) (a Ca precursor), and 0.277 g of sodium tellurite (Na_2_TeO_3_)—was added to the solution while ensuring the stirring. To remove the liquid portion from the bioactive glass formed, a centrifuge was used for 5 min at 6000 rpm. After the separation, to completely remove the residual nitrates, the powder obtained was thermally treated first in an oven at 60 °C for 48 h, followed by 700 °C for 2 h in a furnace, with a heating/cooling ramp of 5 °C/min. To complete the silanisation of the obtained Te-doped bioactive glass, a solution of TMSPMA at 20% in ethanol was placed in contact with the powder for 24 h.

### 2.3. Methacrylation of Chitosan

Following the precedent works performed by our group [25,45], methacrylation of chitosan was performed by dissolving 1.5% (*w*/*v*) of chitosan in a solution of 2% (*v*/*v*) acetic acid in water using a 150 mL beaker. The solution was made homogeneous by increasing the temperature to 50 °C while maintaining constant stirring. Once satisfactory homogeneity was reached, the MA was added dropwise while maintaining the same settling following a ratio of 1:20 between amino glucose moieties and MA. The reaction was maintained at these conditions for 4 h. After that, the solution was dialysed with bi-distilled water for 4 days employing a cellulose membrane (Mw = 14 kDa). When the process was over, the entire product was freeze-dried until all the liquid solvent was evaporated, and a cotton-like product was obtained.

### 2.4. Hydrogel Preparation

**Figure 1:**
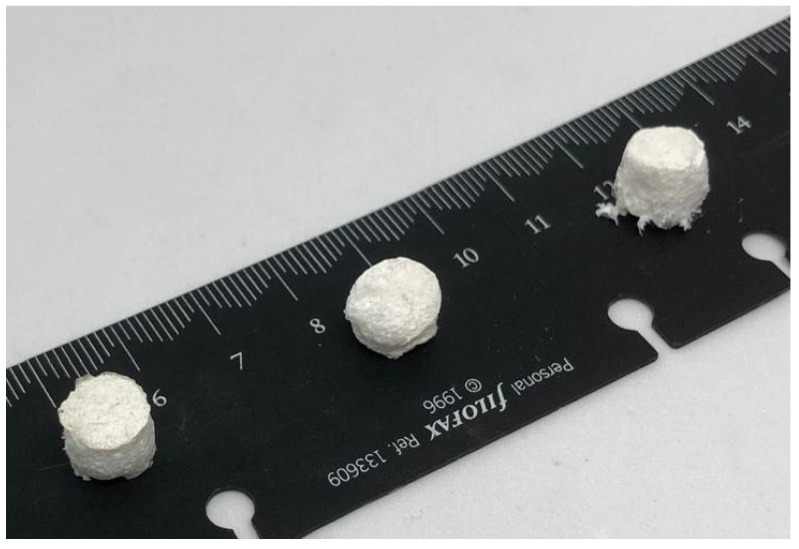
Hydrogels obtained after lyophilisation containing different concentrations of BG-Te-Sil bioactive glass and photocured. From left to right: MCHIT, MCHIT3, and MCHIT5.

Hydrogels were prepared by utilising the methacrylated chitosan (MCHIT) that was previously prepared. A total of 1.5% (*w*/*v*) of MCHIT was dissolved into a slightly acidic solution (2% (*v*/*v*) acetic acid in water) for 2 h at 50 °C in a 150 mL beaker. Once the MCHIT was solubilised, different amounts of previously synthesised BG-Te-Sil were added to the resin mixture and stirred for one hour. The three-mixture compositions are reported in Table 1. Then, 2 phr (parts per hundred resin) of the photoinitiator, 2-Hydroxy-4′-(2-hydroxyethoxy)-2-methylpropiophenone, was dissolved while maintaining the same conditions for an additional hour. The mixture obtained was mixed through an ARE-310 Thinky Mixer (Tokyo, Japan) for 20 min. The hydrogels were cured by pouring them into silicon moulds with a dimension of 10 mm in diameter × 10 mm in height for 3 min under a Dymax lamp at 145 mW/cm^2^ (Wiesbaden, Germany). The UV light was centred around 365 nm. The hydrogel compounds obtained were freeze-dried again, obtaining a white cotton-like product, visible in Figure 1.

### 2.5. FTIR Spectroscopy

Fourier-transform infrared spectroscopy was performed to monitor the presence and consumption of the acrylate groups in MCHIT before and after UV curing through a Thermo Scientific Nicolet iS50 FTIR spectrometer (Thermo Fisher Scientific, Milano, Italy) equipped with a diamond crystal ATR accessory. The experiments were performed on freeze-dried hydrogels. ATR spectra were collected with a resolution of 4 cm^−1^ in the 4000–600 cm^−1^ range.

### 2.6. Field-Emission Scanning Electron Microscopy (FESEM)

Scanning electron microscopy (SEM, JEOL, JCM-6000 plus, Milan, Italy), with an accelerating voltage set at 15.0 kV, was used to observe the surface of the inner hydrogel structure and to investigate the interaction and the dispersion between the polymeric matrix and the reinforcing phase. To observe the samples, they were broken after immersion in liquid nitrogen, then coated with a Platinum layer using a metalliser for 20 s.

### 2.7. Mechanical Tests

Compression tests were performed on the swelled hydrogels placed into a solution of PBS for 24 h to simulate an in vivo test. The machine corresponded to an MTS QTestTM/10 Elite, MTS System Corporation, Eden Prairie, MN, USA, combined with measurement software (TestWorks^®^ 4, version 4.1 MTS System Corporation). The translation speed was set to 5 mm/min with a 10 N cell, using samples with an average diameter of 10 mm and height of 10 mm. In total, 5 samples were tested to calculate the average elastic modulus, determined in the elastic region of the curve.

### 2.8. Photorheology and Rheology Experiments

Rheology tests were performed while irradiating the formulation with UV light by means of an Antoon Paar MC 302 rheometer (Graz. Austria) in a parallel plate (2.5 cm diameter) setup, conducted to evaluate the effect of the incorporation of BG-Te-Sil particles on the viscosity and flow behaviour. The lower plate corresponded to a quartz plate that allowed the permeation of the UV light to ensure the observation of the UV curing process during the irradiation time. The light was activated after one minute at the start of the test. The same analysis was conducted only by changing the lower plate with a metal plate to determine the viscosity of the formulations in a variable shear rate from 0.1 to 1000 s^−1^. The gap between the plates was set to 3 mm for all measurements.

### 2.9. Gel Content Test and Swelling Experiment

Gel content was determined by immersing the dried hydrogels in 5 mL of bi-distilled water to determine the amount of monomer that still had not reacted after the photocuring process. The immersion lasted 24 h, and then, for the subsequent 24 h, the hydrogel was removed from the solution and left to dry in an oven at 50 °C to ensure complete evaporation of the residual water. After the process, the sample was weighed again, and the gel% was calculated in accordance with the following formula:

$$Gel\;content\;\left(\%\right)=\left(\frac{W_{f}}{W_{i}}\right)\times 100$$

The swelling test was performed by placing the dry sample into 5 mL of bi-distilled water. The sample was weighed again at different times up to 24 h but before the weighing, the excess water on the hydrogel surface was removed to ensure only the absorbed water was weighed.

### 2.10. Biological Evaluation of the Hydrogel Samples

#### 2.10.1. Antibacterial Activity Assessment

To evaluate the antibacterial activity of the bio-based matrix derived from methacrylated chitosan reinforced with BG-Te-Sil, a Gram-positive pathogen—multi-drug-resistant *Staphylococcus aureus* (MDR *S. aureus*, ATCC 43300, American Type Culture Collection, Manassas, VA, USA)—was chosen due to its relevance in orthopaedic infections [46,47]. According to the manufacturer’s instructions, Lauria Bertani (LB) broth/agar (Thermo Fisher Scientific, Milan, Italy) was used as the culture medium for bacterial growth at 37 °C.

A fresh subculture was prepared before each experiment, and bacterial growth was monitored by measuring the optical density at 600 nm (OD600 nm). The desired bacterial concentration (1 × 10^5^ colony-forming unit (CFU)/mL) was achieved by diluting the bacterial culture in fresh LB broth. To evaluate the direct antibacterial effect of the hydrogels, 1 mL of the bacterial suspension was dropwise added onto each sample and allowed to adsorb. After 24 h of incubation at 37 °C, the metabolic activity and viable bacterial colony counts on the samples were assessed using the colourimetric AlamarBlue^TM^ assay (Invitrogen, Milan, Italy) and CFU counting, respectively [48]. MCHIT without BG-Te-Sil was considered as a control sample, and the metabolic activity results for MCHIT3 and MCHIT5 were normalised with the result for the control. To quantify the viable bacterial cells attached to the hydrogels, cells were detached by adding 0.5% (*v*/*v*) Tween 80 (Sigma-Aldrich, Milan, Italy) and incubating for 30 min. This was followed by mechanically disrupting the hydrogels, transferring the mixture into 15 mL tubes, and subjecting them to sonication for 5 min, repeated three times with 20-s vortexing intervals. As described in [48], six 10-fold serial dilutions were prepared. From each serial dilution, 20 µL was spotted onto LB agar plates; after 24 h of incubation, the CFU number was calculated. Finally, the morphology of bacterial cells and aggregates within the hydrogel samples was observed by SEM (JSM-IT500, JEOL, Fukuoka, Japan) [48].

#### 2.10.2. Cytocompatibility Evaluation

To evaluate the cytocompatibility of the bio-based hydrogel matrix with BG-Te-Sil for human cells, mesenchymal stem cells derived from bone marrow (hBMSCs, PromoCell, C-12974) were used as a model for tissue regeneration, particularly bone regeneration. Dulbecco’s modified eagle medium (DMEM; Thermo Fisher, Milan, Italy), supplemented with 15% fetal bovine serum (FBS; Sigma Aldrich, Milan, Italy) and 1% antibiotics (penicillin and streptomycin; Sigma-Aldrich, Milan, Italy) was used as the culture medium. Cells were incubated at 37 °C with 5% CO_2_.

For direct seeding within the hydrogels, 100 µL of a mixture containing collagen (3 mg/mL; PureCol^TM^-S, Collagen Standard Solution, Sigma-Aldrich, Milan, Italy) and a cell suspension (2 × 10^6^ cells per sample) was prepared and applied dropwise onto the hydrogel samples. The samples were incubated at 37 °C for 24, 48, and 72 h. At each time point, metabolic activity, cell viability, and morphology were assessed using the colourimetric AlamarBlue^TM^ assay (Invitrogen, Milan, Italy), doubled fluorescent staining with live/dead reagent (Live/Dead Viability/Cytotoxicity Kit for mammalian cells, L3224, Invitrogen, Milan, Italy) plus NucBlue^TM^ Live Cell Stain ReadyProbes^TM^ reagent (R37605, Invitrogen, Milan, Italy), and standard Hematoxylin and Eosin (H&E; Sigma-Aldrich, Milan, Italy) staining, respectively, as explained in the literature [38,49]. MCHIT was considered as a control sample, and the metabolic activity results of MCHIT3 and MCHIT5 were normalised with the result for the control. Three-dimensional fluorescent images demonstrating cells’ distribution within the hydrogel samples were captured using a confocal microscope (TCS SP8 LIGHTNING, Leica, Wetzlar, Germany). Hematoxylin and Eosin images were captured by the brightfield microscope (EVOS FLoid Cell Imaging microscope, Thermo Fisher Scientific, Milan, Italy) with 20× magnification.

#### 2.10.3. Anti-Inflammatory Characteristics

To confirm the anti-inflammatory properties of Te-Sil in the 3D scaffold samples, inflammation was chemically induced using hydrogen peroxide (H_2_O_2_; Sigma-Aldrich, Milan, Italy), and the protective effect of BG-Te-Sil on hBMSCs was evaluated. As described in the previous section, human cells were seeded into hydrogel samples and incubated for 24 h to allow cell spreading. Inflammation was then induced by adding H_2_O_2_ at a concentration of 300 µM per sample for 7 days [50]. Gene expression of pro-inflammatory markers (purchased from Integrated DNA Technologies, USA)—Interleukin-1 beta (IL-1β), Tumour necrosis factor-alpha (TNF-α), Interferon-gamma (IFN-γ), Prostaglandin-E synthase (PGES-2), and the anti-inflammatory marker Interleukin-10 (IL-10)—was analysed using quantitative real-time PCR (qRT-PCR; RNeasy plus Mini Kit, QIAGEN 74134, The Netherlands) [51,52]. Gene expression fold-change was quantified using the 2^−ΔΔCt^ method, with Glyceraldehyde 3-phosphate dehydrogenase (GAPDH) as the housekeeping gene for normalisation. The forward and reverse primers are listed in Table A1 (Appendix A).

#### 2.10.4. Statistical Evaluation

The results were compared through statistical analysis, conducted by SPSS software (v.20.0, IBM, Armonk, NY, USA). At first, the normal distribution and homogeneity of variance of the data were checked. Then, the differences between groups were clarified using a one-way ANOVA and Tukey’s test as the post hoc analysis. A *p* < 0.05 is considered a statistically significant difference and is indicated by an asterisk (*).

## 3. Results and Discussion

### 3.1. Methacrylation Bio-Based Monomer

**Figure 2:**
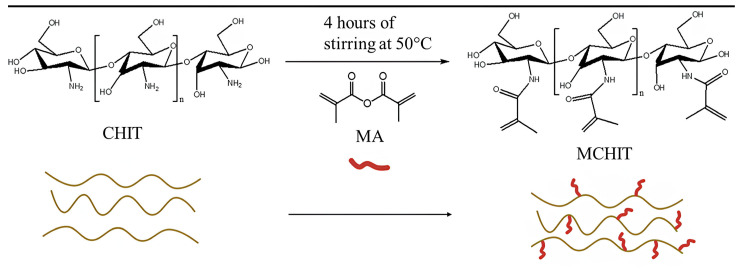
Methacrylation reaction of chitosan.

Chitosan was functionalised through the grafting of methacrylic groups (methacrylation reaction) following a previous method already used in our group to have the bio-based monomer ready for the photocuring process. The methacrylation scheme is reported in Figure 2.

### 3.2. Characterisation of MCHIT

A comprehensive characterisation was performed before and after UV curing using FTIR and photorheology to verify the correct methacrylation of chitosan and study the photocuring process of the hydrogel. All resin compositions were tested to assess the potential interference of bioactive glasses. The irradiation time was set to 6 min at an intensity of 145 mW/cm^2^ using a Dymax lamp. This duration was required for the formulation to reach consistency and be removable from the silicone mould used.

**Figure 3:**
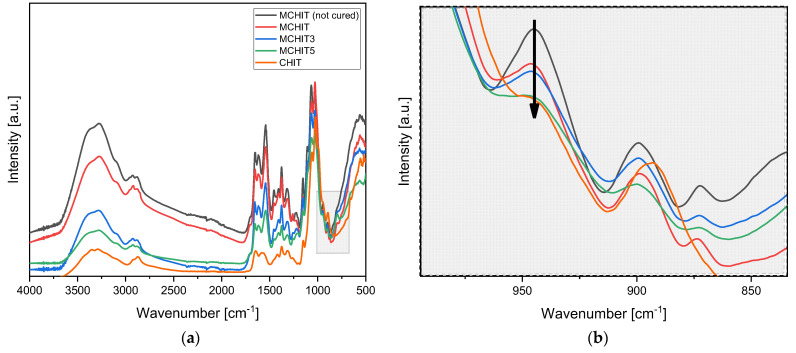
(**a**) Overall FTIR spectra of all the tested formulations; (**b**) magnification of (**a**) in the range of 1100 to 830 cm^−1^. The black arrow represent the epoxy peak decrease after curing.

In Figure 3, the FTIR spectra of all tested formulations are reported. First, the presence of acrylate groups around 945 cm^−1^ in the pristine MCHIT shows the successful methacrylation of chitosan (CHIT), especially when compared to the starting CHIT, which does not show the acrylate peak. Moreover, the FTIR spectra clearly indicate that the reaction occurs in all formulations reinforced with hydrogels. This is evidenced by the fact that MCHIT, which was not photocured as it was not exposed to UV light, contains the highest amount of acrylate groups. In contrast, all other formulations exposed to 365 nm UV light exhibit a decrease in the acrylate group signal at 945 cm^−1^, confirming that the photopolymerisation reaction has occurred. The lower prominence of the acrylate groups is not only due to their actual reduction in the reinforced formulations MCHIT3 and MCHIT5 but also due to the normalisation process during data analysis. Specifically, as the amount of bioactive glass increases, the highest peak used for normalisation corresponds to the bioactive glass, making the acrylate peaks appear less prominent than the other cured formulations.

**Figure 4:**
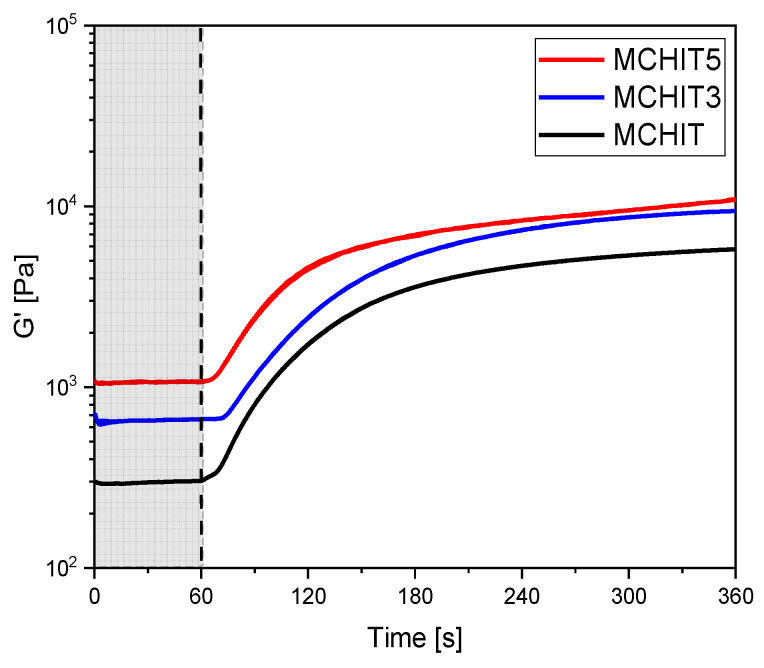
Photorheology graph. The UV light was directed to the sample 60 s after the start of the measurement (end of the gray region).

To confirm the photocuring process studied with FTIR, all formulations underwent a photorheology experiment. The experiment involved irradiating the liquid resin under UV light while monitoring its rheological behaviour. The results of the test are reported in Figure 4.

After switching on the UV light, which occurred 60 s after the start of the trial, the formulation began the crosslinking process. The process, with similar behaviour for all formulations, exhibited a slight delay of 10 s. However, once initiated, the reaction was completed within 180 s under a UV light intensity of 145 mW/cm^2^ at 365 nm.

**Figure 5:**
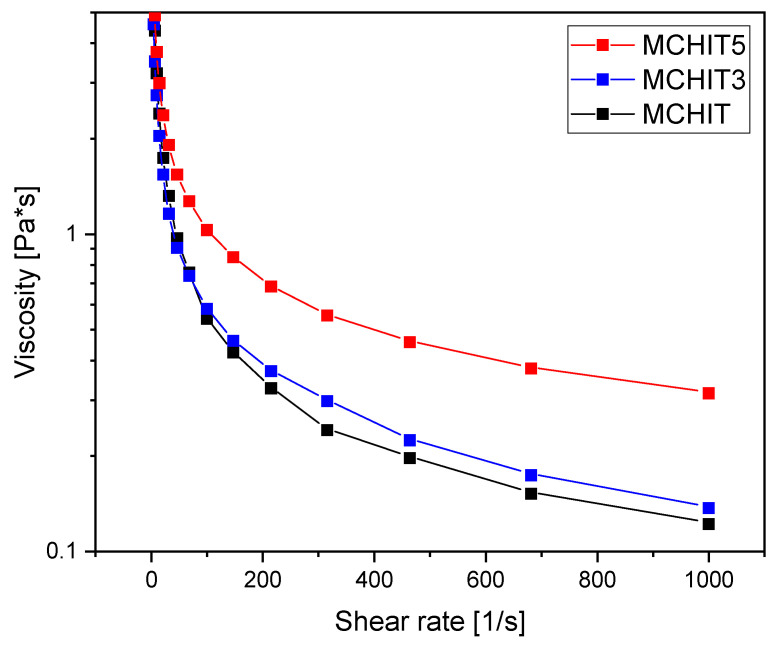
Rheology curves obtained with a shear rate of 0 to 1000 s^−1^.

As expected, the initial G’ in the shadow zone, representing the modulus of the resin before the curing reaction, was lower for MCHIT. In fact, in the reinforced formulations, the modulus increased gradually due to the ceramic nature of the bioactive glass, which made the formulation more viscous. To verify this, a viscosity test was performed under varying shear rates. As reported in Figure 5, the results confirm that while MCHIT and MCHIT3 exhibited similar behaviour with increasing shear rate, MCHIT5 showed a significant increase in viscosity.

Once the hydrogel curing process was confirmed, we proceeded with hydrogel manufacturing. A silicone mould with a height of 1 cm and a diameter of 1 cm was used to contain the resin precursor.

**Figure 6:**
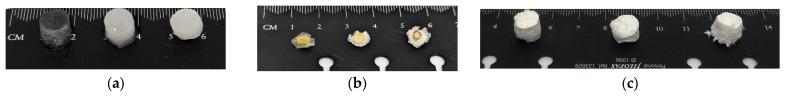
(**a**) Hydrogels obtained after the photocuring process; (**b**) air-dried hydrogels; (**c**) freeze-dried hydrogels.

After curing, the samples were removed from the silicone mould and placed directly into the lyophiliser to dry them while maintaining their shape. The direct drying method under air in a fume hood was avoided because, as visually reported in Figure 6, it results in structural compression and loss of the original shape.

### 3.3. Hydrogel Characterisation

A gel percentage experiment was conducted to verify that the hydrogels were fully cured. After being soaked in distilled water overnight, the samples were weighed, and the gel percentage was recorded. The results are presented in Table 2.

The results show a full gel percentage, indicating the absence of residual uncured resin. This suggests that during in vitro testing, no acrylate groups are released into the environment, thereby eliminating cytotoxicity concerns related to unreacted species.

**Figure 7:**
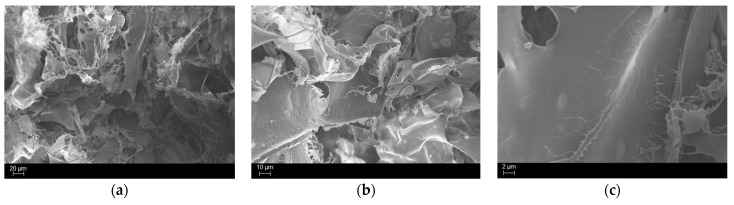
SEM images, at different magnifications, of MCHIT samples. Scale bar of (**a**) is set to 20 µm; scale bar of (**b**) is set to 10 µm; scale bar of (**c**) is set to 2 µm.

**Figure 8:**
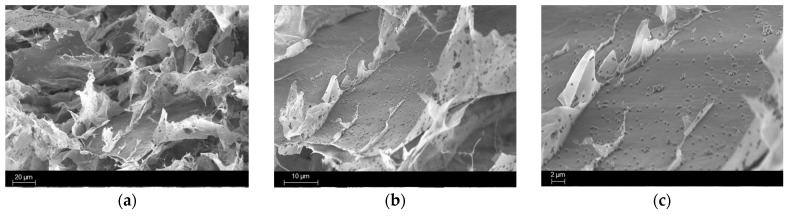
SEM images, at different magnifications, of MCHIT3 samples. Scale bar of (**a**) is set to 20 µm; scale bar of (**b**) is set to 10 µm; scale bar of (**c**) is set to 2 µm.

**Figure 9:**
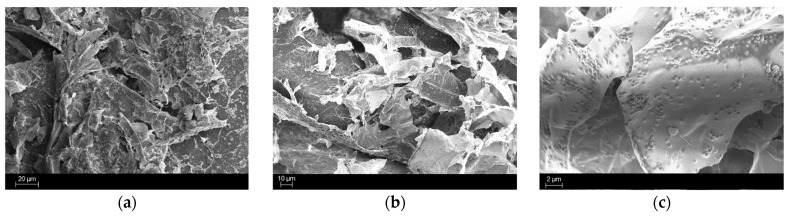
SEM images, at different magnifications, of MCHIT5 samples. Scale bar of (**a**) is set to 20 µm; scale bar of (**b**) is set to 10 µm; scale bar of (**c**) is set to 2 µm.

After confirming the absence of unreacted resin within the cured hydrogels, the structure was analysed using FESEM. The sample preparation involved freezing in liquid nitrogen, followed by splitting the sample in half and coating it with a layer of platinum to enhance conductivity. All the images collected are reported in Figure 7, Figure 8 and Figure 9. The figures represent all the samples prepared with the various bioactive glass percentages.

From Figure 7, Figure 8 and Figure 9, it is evident that adding BG particles within the formulation leads to a desired uniform dispersion in the matrix. Moreover, no voids are present in the structure, indicating optimal attachment to the polymeric phase. This is attributed to the silanisation process, which enhances the compatibility between the reinforcement phase and the matrix through the attachment of aliphatic chains. The MCHIT5 samples present more particles distributed along the surface but despite their increased concentration, only a tiny cluster of aggregated particles are present, maintaining a uniform dispersion.

**Figure 10:**
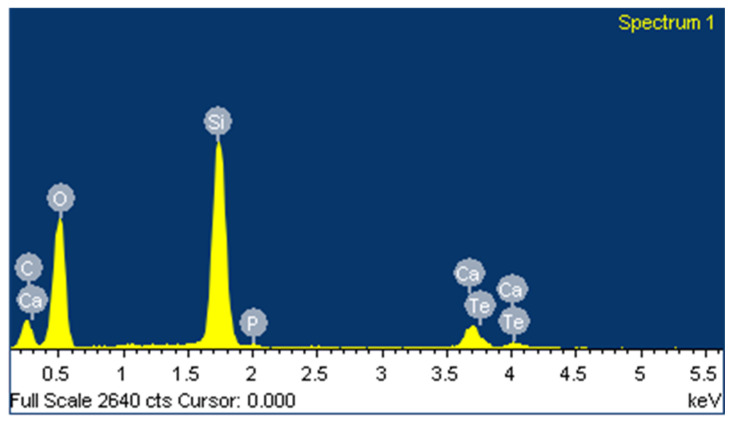
EDS measurement performed on the BG-Te particle to identify the main elements present.

To confirm the presence of Te in the bioactive glass particles, EDS analysis was performed on the same sample setup but focusing on a single BG particle, as shown in Figure 10. From the figure, it is clear that Te was successfully incorporated into the BG, as indicated by its peak in the spectrum.

**Figure 11:**
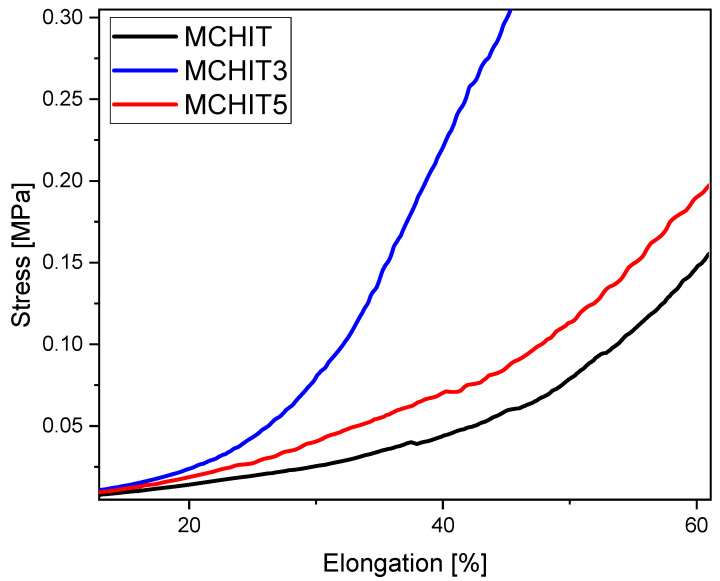
Compression tests performed on all formulations. The graph shows the mean values obtained from five independent measurements.

After determining the optimal interface between the matrix and the reinforcing phase, the mechanical properties of the hydrogels were studied. This analysis was conducted on wet hydrogels previously immersed in PBS, focusing on their compressive mechanical properties. The results are presented in Figure 11 and Table 3. The findings demonstrate the reinforcement effect provided by the bioactive glass within the formulation. When 30 phr of BG is used, the compressive modulus increases drastically by almost 200% of its initial value as the ceramic component begins to resist external forces. However, increasing the BG content to 50 phr leads to a decrease in the compressive modulus, likely due to local particle aggregation, which disrupts the crosslinked structure and reduces the dispersion of the reinforcement phase, as confirmed by other authors in similar studies [53,54]. As a result, the matrix is no longer well connected, diminishing the synergistic effect, although the mechanical properties still show improvement compared to the reference sample.

### 3.4. Biological Evaluation of the Hydrogel Samples

This study builds upon our previous work [44], which involved producing 3D-printed acrylated soybean oil scaffolds reinforced with BG-Te and BG-Te-Sil. These scaffolds demonstrated reduced bacterial metabolic activity and aggregation while remaining biocompatible, supporting hBMSC cell attachment and spreading. In the current work, by confirming the role of silanisation in improving the interface between the reinforcement phase and the matrix, a 3D bio-based hydrogel scaffold model with a height of 1 cm was developed using methacrylated chitosan, reinforced with BG-Te-Sil. To evaluate their potential for tissue implant applications, two sample groups were prepared with reinforcement of 30 phr and 50 phr, referred to as MCHIT3 and MCHIT5, respectively.

### 3.5. Antibacterial Activity Assessment

**Figure 12:**
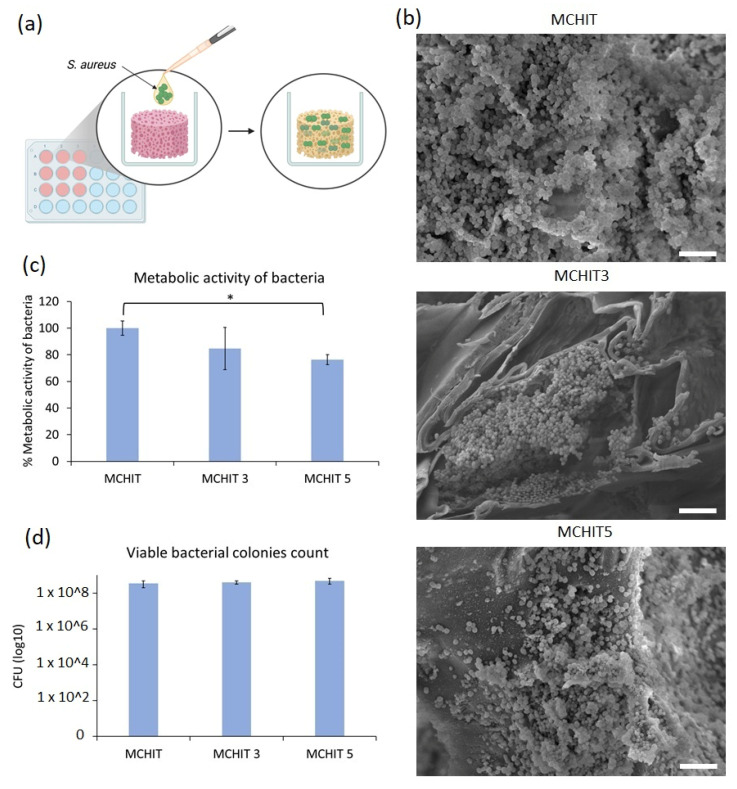
Antibacterial activity of 3D hydrogel scaffolds made of methacrylated chitosan reinforced with 30 phr BG-Te-Sil (MCHIT3) and 50 phr BG-Te-Sil (MCHIT5) against MDR *S. aureus* after 24 h of incubation: (**a**) schematic diagram of infection samples with bacteria; (**b**) bacterial metabolic activity, the results of MCHIT3 and MCHIT5 were normalised with the MCHIT (control) result, * indicates *p* < 0.05; (**c**) viable bacterial colony count (CFU) adhered to and within the samples; (**d**) SEM images, scale bar = 5 µm.

The pathogenicity of multi-drug-resistant *S. aureus* in tissue implant infections and the challenges posed by its biofilm formation are well studied [55]. To evaluate the antibacterial properties of the samples, 1 mL of bacterial suspension was applied to the 3D hydrogel scaffolds, allowing the bacteria to adsorb and infiltrate the hydrogels (Figure 12a). After 24 h of incubation and transferring the hydrogels to a new 24-well plate, the bacterial metabolic activity was measured by adding the colourimetric AlamarBlue^TM^ reagent directly to the wells containing the samples. To quantify viable bacterial cells adhered to and within the hydrogels, the cells were detached and counted, as described in detail in the corresponding Materials and Methods Section. The results are presented in Figure 12b,c. A slight reduction in bacterial metabolic activity was observed in MCHIT5 samples compared to MCHIT (the control), with statistical significance at *p* < 0.05, indicated by * (Figure 12b). However, the CFU numbers did not significantly differ between MCHIT3, MCHIT5, and the control (Figure 12c). Additionally, SEM images revealed extensive biofilm formation on the MCHIT hydrogels; in contrast, MCHIT3 and MCHIT5 showed biofilm localised within internal spaces, which is more evident in the SEM images for the MCHIT3 samples (Figure 12d). Our previous study indicated that 3D-printed scaffolds reinforced with BG-Te outperformed BG-Te-Sil ones in reducing bacterial aggregates [44]. According to the literature, Te exhibits antibacterial effects by entering bacterial cells and inhibiting key enzymes that play essential roles in bacterial growth and biofilm formation [56]. Therefore, incorporating BG-Te-Sil into MCHIT3 and MCHIT5 may have decreased the availability of Te for bacterial cells. However, MCHIT5, which contained 50 phr BG-Te-Sil, significantly reduced bacterial metabolic activity. As shown in Figure 8 and Figure 9, the number of particles in the SEM images was more than that of MCHIT (the control) and MCHIT3. This increased number of particles, and thus higher BG content, may explain the lower bacterial metabolic activity and aggregates observed in MCHIT5. Enhancement of the antibacterial activity of these 3D hydrogel scaffolds needs more investigation and further modification without decreasing the mechanical properties of the hydrogels.

### 3.6. Cytocompatibility Evaluation

**Figure 13:**
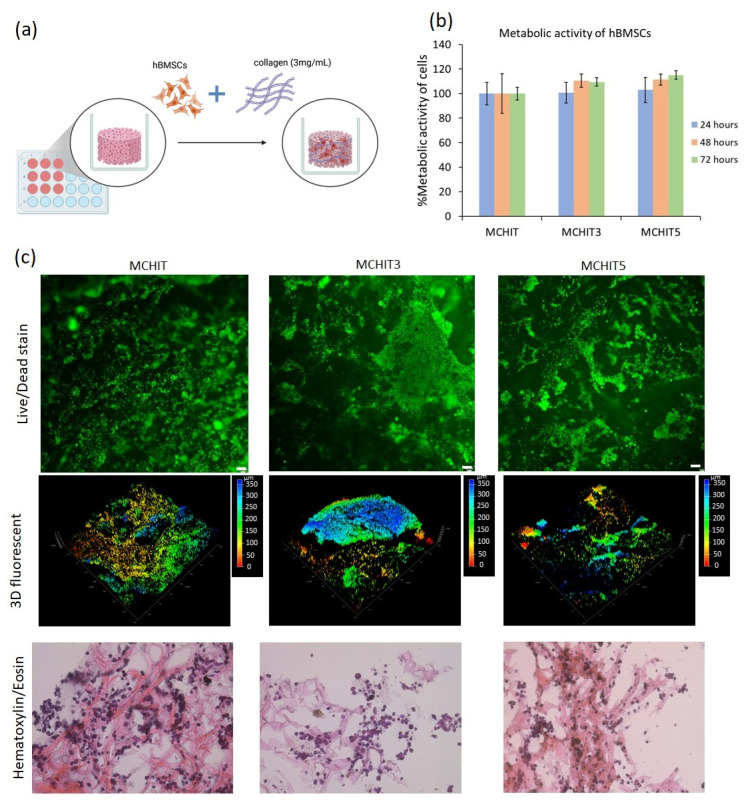
Cytocompatibility evaluation of 3D hydrogel scaffolds made of methacrylated chitosan reinforced with 30 phr BG-Te-Sil (MCHIT3) and 50 phr BG-Te-Sil (MCHIT5) towards hBMSC cells after 24–72 h of incubation: (**a**) Schematic diagram illustrating the seeding of cells within the hydrogels using collagen (3 mg/mL). (**b**) Metabolic activity of the cells at each time point, with the results of MCHIT3 and MCHIT5 normalised to those MCHIT (control). (**c**) Top panel: live/dead fluorescent staining, scale bar = 50 µm; middle panel: 3D fluorescent images; the measure scales (µm) demonstrate the thickness of cell aggregations within the hydrogel; bottom panel: histological staining (Hematoxylin/Eosin). Images (captured with a bright field microscope with magnification of 20×) were captured after 72 h of incubation of the cells within the hydrogels.

**Figure A1:**
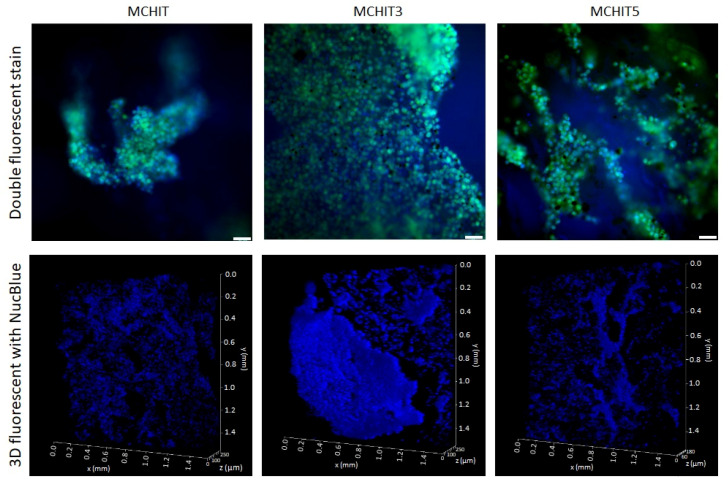
Top panel: Double fluorescent staining after 72 h of seeding the hBMSCs within the 3D hydrogel scaffolds—live/dead, which indicated the viable cells, and NucBlue, which indicated the nuclei of the cells, scale bar = 50 µm. Bottom panel: 3D fluorescent images demonstrating the cells’ distribution using NucBlue stain.

For the cytocompatibility assessment of the 3D hydrogel scaffolds reinforced with BG-Te-Sil (MCHIT3 and MCHIT5), hBMSC cells—representative of tissue regeneration—were seeded within the hydrogels using collagen (3 mg/mL) to ensure that the cells remained attached inside the hydrogels (with a height of 1 cm) and did not pass through them (Figure 13a). At each time point (24, 48, and 72 h), the metabolic activity was analysed using the AlamarBlue reagent, and after 72 h, the viability and the distribution of the hBMSC cells within the hydrogels were assessed using live/dead and NuclBlue fluorescent staining, 3D fluorescent imaging, and Hematoxylin/Eosin staining; the results are presented in Figure 13b,c and Figure A1. As shown in Figure 13b, no significant differences in the metabolic activity were observed between MCHIT3 and MCHIT5 in comparison to MCHIT (the control) at all time points. The metabolic activity results of MCHIT3 and MCHIT5 were normalised to those of MCHIT. According to the fluorescent staining, the majority of the cells within the hydrogels were alive (stained green, Figure 13c, top panel), and the cells were evenly distributed throughout the samples (Figure 13c, middle panel). NucBlue reagent stained the nucleus of cells, and Figure A1, Top panel (Appendix A.1.2) shows the merging double fluorescent staining; additionally, Figure A1b, Bottom panel (Appendix A.1.2) presents the 3D NucBlue fluorescent imaging, which was captured using Z-stack photos by a confocal microscope. In the Hematoxylin/Eosin staining, the cells appeared circular and stained violet (dark red), while the matrix of the hydrogels was distinguishable by its strand-like shapes and light red colour (Figure 13c, bottom panel; magnifications are 20×). These results confirmed the cytocompatibility and uniform distribution of hBMSCs within the bio-based hydrogel matrix reinforced with BG-Te-Sil. Our previous work [44] demonstrated the safety of BG-Te and BG-Te-Sil in the 3D-printed acrylated soybean oil scaffolds towards hBMSCs, which were seeded on the samples’ surfaces after 24 h. As described in the previous section, this study was built upon that work, and here, the cytocompatibility of the BG-Te-Sil was applied within the 3D hydrogel scaffolds made of methacrylated chitosan with a height of 1 cm. Additionally, our previous work showed that the scaffolds containing 30 phr of BG not only had suitable characteristics for 3D printing but also demonstrated higher cytocompatibility for human cells in comparison with the control (without BG) and 10 phr of BG samples [38]. Therefore, in this study, the hydrogels reinforced with 30 phr and 50 phr BG-Te-Sil were compared with each other and with control samples to evaluate their safety towards human cells.

### 3.7. Anti-Inflammatory Characterisation

**Figure 14:**
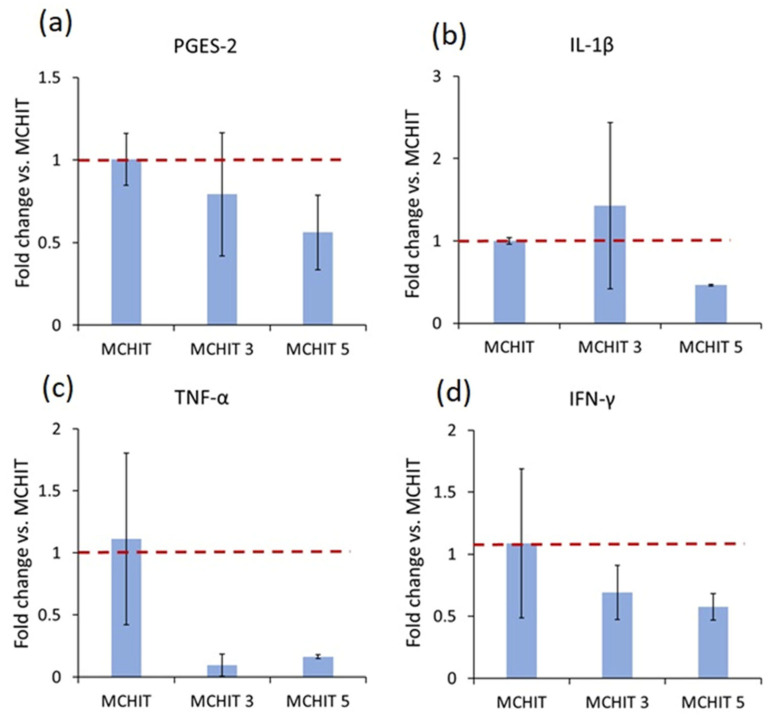
Fold changes in the expression of genes encoding pro-inflammatory responses were calculated using the 2^−ΔΔCT^ method. The red line represents a fold-change value of 1, where any value above 1 is considered upregulated, and any value below 1 is considered downregulated: (**a**) Prostaglandin-E Synthase 2 (PGES-2); (**b**) Interleukin-1 beta (IL-1β); (**c**) Tumour necrosis factor-alpha (TNF-α); (**d**) Interferon-gamma (IFN-γ). Gene expression of Glyceraldehyde 3-phosphate dehydrogenase (GAPDH) was used as the housekeeping gene.

Bone is a continuously remodelled tissue, and maintaining equilibrium between the mineralising activity of osteoblasts and the resorptive function of osteoclasts is crucial. This balance can be disrupted by some factors, such as hormones, cytokines, and reactive oxygen species (ROS) [57]. The anti-inflammatory properties of Te have been well known for many years, and a substantial body of literature supports the anti-inflammatory effects of Te and TeO_2_ [30,33,58]. One of the objectives of using BG-Te-Sil was to enhance the biocompatibility of the bio-based hydrogel made of methacrylated chitosan by combining the cytocompatibility offered by 30 phr and 50 phr BG, the anti-inflammatory impact of Te, and strong mechanical reinforcement through silanisation. To evaluate the anti-inflammatory effect of the BG-Te-Sil in MCHIT3 and MCHIT5, after seeding the hBMSCs within the hydrogels—as described in detail in Section 3.6—the inflammation was chemically induced by adding 300 µM of H_2_O_2_ for 7 days. At the end of the incubation period, changes in the expression of genes encoding pro- and anti-inflammatory markers (IL-1β, TNF-α, IFN-γ, PGES-2, and IL-10) were evaluated using qRT-PCR, and the fold changes were calculated by the 2^−ΔΔCt^ method. The red lines indicated thresholds for gene regulation: values of 2^−ΔΔCt^ greater than 1 were considered upregulated, while values less than 1 were considered downregulated [59]. As shown in Figure 13, exposure of the hBMSCs within MCHIT3 and MCHIT5 to H_2_O_2_ for 7 days resulted in the downregulation of genes encoding pro-inflammatory responses, particularly TNF-α and IFN-γ (Figure 14c,d). Additionally, a noticeable reduction in the expression of PGES-2 and IL-1β (Figure 14a,b) was observed in the MCHIT5 samples containing 50 phr BG-Te-Sil. As mentioned earlier, the anti-inflammatory effect of Te is attributed to its ability to scavenge reactive oxygen species (ROS) and inhibit the activation of transcription factors that regulate pro-inflammatory genes. The anti-inflammatory mechanism is mediated by leached Te species, released as Te(IV), which donate electrons to neutralise H_2_O_2_ and -OH, cycling through lower oxidation states [30,33].

Recently, these properties of Te have attracted significant attention from researchers developing biomaterials to enhance biocompatibility. Mathai and Shaji (2023) [60] developed a new titanium implant coating with tellurium dioxide–niobium pentoxide, suitable for bone-replacement surgery applications. These nanocomposite coatings, prepared using low-temperature, low-cost techniques, demonstrated strong anti-inflammatory effects as well as antibacterial activity against both Gram-positive and Gram-negative bacteria [60]. Similarly, Hu et al. (2024) constructed oral tellurium-containing drug carriers by complexing tellurium-containing polycarbonate with cisplatin, aiming to target intratumoural bacteria and control inflammation at tumour sites [61]. Based on these reports, the current study aimed to improve the physico-chemical and biocompatibility properties of 3D bio-based hydrogel scaffolds reinforced with BG-Te-Sil for tissue implant applications. According to the obtained results, the hydrogel scaffolds were not toxic to human cells, and, thanks to the presence of Te, they caused downregulation of pro-inflammatory gene expression. However, further investigation is needed to enhance their antibacterial activity, particularly against multi-drug-resistant bacteria.

## 4. Conclusions

In this study, a hydrogel system reinforced with silica-based and silanised bioactive glasses doped with tellurium was developed, utilising methacrylated chitosan (MCHIT) as the polymer matrix. The results demonstrated the successful methacrylation of chitosan, enabling efficient photopolymerisation. FTIR and photorheology confirmed the complete crosslinking of the system, while gel content tests evidenced the absence of uncured hydrogel, ensuring the chemical stability of the material in SBF.

Incorporating BG-Te-Sil significantly improved the mechanical properties of the hydrogel, with an increase in modulus, particularly in the MCHIT3 samples. However, a further increase in BG concentration in MCHIT5 led to a slight reduction in mechanical performance, likely due to local particle aggregation. FESEM images confirmed an optimal dispersion of BG particles within the polymeric matrix without the formation of empty spaces or structural defects, highlighting the effectiveness of the silanisation process in improving the polymer–reinforcement interface.

The biological evaluation of the 3D bio-based hydrogel scaffolds, made of methacrylated chitosan and reinforced with 30 phr and 50 phr BG-Te-Sil (referred to as MCHIT3 and MCHIT5, respectively), demonstrated that human bone-marrow mesenchymal stem cells (hBMSCs) seeded within these 1-centimeter-high samples remained viable after 72 h of incubation and were uniformly distributed throughout the hydrogels. Furthermore, exposure of the hBMSCs to chemically induced inflammation using H_2_O_2_ for 7 days resulted in the downregulation of the pro-inflammatory genes, particularly TNF-α and IFN-γ, compared to the MCHIT (control) sample. Further investigations are required to enhance the antibacterial activity as a significant reduction in the metabolic activity of the multi-drug-resistant (MDR) *S. aureus* was observed for MCHIT5, which contains 50 phr BG-Te-Sil. It is worth noting that the improvement in the biological properties of the 3D hydrogel scaffolds should be aligned with the optimisation of the matrix’s physico-chemical and mechanical characteristics.

Overall, the obtained results suggest that our system represents a promising option for tissue engineering applications, combining biocompatibility, biological functionalities, efficient photopolymerisation, and enhanced mechanical properties.

## Figures and Tables

**Table 1:** Names attributed to the different formulations based on the amount of bioactive glass Te-doped within the composition. The phr (parts per hundred resin) is calculated from the dry quantity of methacrylated chitosan.

Name	BG-Te-Sil Amount [phr]
MCHIT	0
MCHIT3	30
MCHIT5	50

**Table 2:** Gel content results after soaking the sample in water for 24 h.

Sample Name	%Gel
MCHIT	99.8 ± 0.1
MCHIT3	99.8 ± 0.1
MCHIT5	99.9 ± 0.0

**Table 3:** Results obtained from the compression tests. The compressive modulus was evaluated in the linear region of the curve.

Sample Name	Compressive Modulus [KPa]
MCHIT	87 ± 25
MCHIT3	246 ± 51
MCHIT5	80 ± 34

## Data Availability

The original contributions presented in this study are included in the article. Further inquiries can be directed to the corresponding author.

## Appendix A

### Appendix A.1

#### Appendix A.1.1. Table A1 List of the Forward and Reverse Pro- and Anti-Inflammatory Genes

To evaluate the anti-inflammatory response, hBMSCs were seeded within the 3D hydrogel scaffolds and exposed to 300 µM H_2_O_2_ for 7 days to induce chemical inflammation. After that incubation period, changes in the expression of pro- and anti-inflammatory genes were calculated using 2^−ΔΔCT^ methods, with MCHIT serving as the control sample. Table A1 presents the forward and reverse primer sequences for the selected genes involved in pro- and anti-inflammatory responses: PEGS-2, TNF-α, IFN-γ, IL-1β, and IL-10. GAPDH was used as the housekeeping gene.

**Table A1:** List of forward and reverse primers.

Gene	Abbreviation	Forward (5′-3′)	Reverse (5′-3′)
Prostaglandin-E Synthase 2	PGES-2	5′-ACGACTTGGAGACCATCTA-3′	5′-GAAGACCAGGAAGTGCATC-3′
Interleukin 10	IL10	5′-CTCCAAGAGAAAGGCATCTAC-3′	5′-CACCCTGATGTCTCAGTTTC-3′
Tumour Necrosis Factor-alpha	TNFα	5′- AGAGGGAGAGAAGCAACTAC-3′	5′-GGGTCAGTATGTGAGAGGAA-3′
Interferon-gamma	IFNγ	5′-CTT GAA TGT CCA ACG CAA AG-3′	5′-CCTCGAAACAGCATCTGAC-3′
Interleukin 1β	IL1β	5′-AAGGCGGCCAGGATATAA-3′	5′-GGGATTGAGTCCACATTCAG-3′
Glyceraldehyde-3-phosphate Dehydrogenase	GAPDH	5′-GTATGACAACAGCCTCAA GAT-3′	5′-GTCCTTCCACGATACCAAAG-3′

#### Appendix A.1.2. Figure A1 Double Fluorescent Staining (Live/Dead Plus NucBlue) and 3D Fluorescent Images

After seeding hBMSCs within the 3D hydrogel scaffolds and incubating them for 72 h, double fluorescent staining was performed using live/dead staining (Live/Dead Viability/Cytotoxicity Kit for mammalian cells, L3224; Invitrogen, Milan, Italy) and NucBlue^TM^ Live Cell Stain ReadyProbes^TM^ reagent (R37605; Invitrogen, Milan, Italy), indicating viable cells within hydrogels in green (with live/dead stain) with the nuclei in blue (with NucBlue). As described in detail in the manuscript, the fluorescent images and 3D fluorescence with NucBlue were captured using a confocal microscope (TCS SP8 LIGHTNING, Leica).
